# The histone and non-histone methyllysine reader activities of the UHRF1 tandem Tudor domain are dispensable for the propagation of aberrant DNA methylation patterning in cancer cells

**DOI:** 10.1186/s13072-020-00366-4

**Published:** 2020-10-23

**Authors:** Robert M. Vaughan, Ariana Kupai, Caroline A. Foley, Cari A. Sagum, Bailey M. Tibben, Hope E. Eden, Rochelle L. Tiedemann, Christine A. Berryhill, Varun Patel, Kevin M. Shaw, Krzysztof Krajewski, Brian D. Strahl, Mark T. Bedford, Stephen V. Frye, Bradley M. Dickson, Scott B. Rothbart

**Affiliations:** 1 Center for Epigenetics, Van Andel Institute, Grand Rapids, MI 49503 USA; 2 Center for Integrative Chemical Biology and Drug Discovery, Division of Chemical Biology and Medicinal Chemistry, UNC Eshelman School of Pharmacy, University of North Carolina At Chapel Hill, Chapel Hill, NC 27599 USA; 3 Department of Epigenetics and Molecular Carcinogenesis, University of Texas MD Anderson Cancer Center, Smithville, TX 78957 USA; 4 Department of Biochemistry and Biophysics, University of North Carolina At Chapel Hill, Chapel Hill, NC 27599 USA

## Abstract

The chromatin-binding E3 ubiquitin ligase ubiquitin-like with PHD and RING finger domains 1 (UHRF1) contributes to the maintenance of aberrant DNA methylation patterning in cancer cells through multivalent histone and DNA recognition. The tandem Tudor domain (TTD) of UHRF1 is well-characterized as a reader of lysine 9 di- and tri-methylation on histone H3 (H3K9me2/me3) and, more recently, lysine 126 di- and tri-methylation on DNA ligase 1 (LIG1K126me2/me3). However, the functional significance and selectivity of these interactions remain unclear. In this study, we used protein domain microarrays to search for additional readers of LIG1K126me2, the preferred methyl state bound by the UHRF1 TTD. We show that the UHRF1 TTD binds LIG1K126me2 with high affinity and selectivity compared to other known methyllysine readers. Notably, and unlike H3K9me2/me3, the UHRF1 plant homeodomain (PHD) and its N-terminal linker (L2) do not contribute to multivalent LIG1K126me2 recognition along with the TTD. To test the functional significance of this interaction, we designed a LIG1K126me2 cell-penetrating peptide (CPP). Consistent with LIG1 knockdown, uptake of the CPP had no significant effect on the propagation of DNA methylation patterning across the genomes of bulk populations from high-resolution analysis of several cancer cell lines. Further, we did not detect significant changes in DNA methylation patterning from bulk cell populations after chemical or genetic disruption of lysine methyltransferase activity associated with LIG1K126me2 and H3K9me2. Collectively, these studies identify UHRF1 as a selective reader of LIG1K126me2 in vitro and further implicate the histone and non-histone methyllysine reader activity of the UHRF1 TTD as a dispensable domain function for cancer cell DNA methylation maintenance.

## Introduction

Ubiquitin-like with PHD and RING finger domains 1 (UHRF1, UniProtKB Q96T88, or Np95 in *M. musculus*, UniProtKB Q8VDF2) is an E3 ubiquitin ligase that binds to histones [1], various modified forms of deoxyribonucleic acid (DNA) [2–4], DNA methyltransferases [5–7], ubiquitin-conjugating enzymes (E2s) [8–10], and a deubiquitinase (DUB) [11]. The combination of these established interactions suggests a major function of UHRF1 is to deposit histone ubiquitination [1, 8, 12]. UHRF1 functions as a DNA methylation maintenance factor, a characteristic that is likely dependent on its catalytic activity toward multiple sites of mono-ubiquitination on histone H3 (UniProtKB, P68431) [8, 13, 14] and PCNA-associated factor (PAF15, UniProtKB Q15004) [15], which are bound by the ubiquitin-interacting motif (UIM) of DNA (cytosine-5)-methyltransferase 1 (DNMT1, UniProtKB P26358) [16–18]. While the oncogenic role of UHRF1 is emerging [19, 20], efforts to antagonize UHRF1 function have been challenging. Thus, a deep understanding of the UHRF1 protein–protein interaction network may reveal novel ways to disrupt its molecular function as a DNA methylation regulator.

An interaction between UHRF1 and DNA ligase 1 (LIG1, UniProtKB P18858) was first identified by tandem affinity purification of a UHRF1 transgene followed by mass spectrometry [11]. This interaction was recently shown to be methyllysine-dependent [21, 22]. The UHRF1 tandem Tudor domain (TTD) binds lysine 9 di- and tri-methylation on histone H3 (H3K9me2/me3) and all three methylation states of lysine 126 on LIG1 (LIG1K126) [21]. The contribution of this non-histone methyllysine-driven interaction to UHRF1-dependent DNA methylation maintenance is unclear. In mouse embryonic stem cells (mESCs), deletion of LIG1 had no effect on DNA methylation measured by mass spectrometry of bulk 5mC, luminometric methylation assay, and reduced representation bisulfite sequencing (RRBS) [21]. However, in this same study, DNA methylation analysis revealed a defect in mESCs in which 32 or 66 residues surrounding lysine 126 on LIG1 (the reported site of LIG1 methylation) were deleted. The contribution of this methyllysine-driven interaction to cancer cell DNA methylation maintenance through UHRF1 has not been considered.

We focused primarily on di-methylation of lysine 126 on LIG1 (LIG1K126me2), as it had the highest affinity of the LIG1K126 methyl orders for full-length UHRF1 and was reported to be the predominant methyl form in several cell types [21]. The amino acids surrounding LIG1K126 resemble those around H3K9, earning LIG1 the title of “histone-mimic”, a term first used to describe short linear motifs shared between histone H3 and viral proteins [23]; these motifs are present in various chromatin-related proteins [24].

In this study, we searched for additional readers of LIG1K126me2 and found that the UHRF1 TTD reads this PTM with striking selectivity over known methyllysine readers. We contrasted the interaction of UHRF1 with LIG1 and H3 and concluded that the UHRF1 plant homeodomain (PHD) drives H3 recognition, whereas the TTD drives the interaction with LIG1. However, treatment of cells with a cell-penetrating LIG1K126me2 peptide, LIG1 transgene overexpression, or stable LIG1 knockdown had no effect on the DNA methylation maintenance function of UHRF1 in several cancer cell lines. Further, using chemical and genetic approaches to reduce lysine methylation, on both histone and non-histone proteins, we found no changes in DNA methylation by query of ~ 850,000 unique CpG probes on the Infinium Methylation EPIC BeadChip array platform. The data presented here, combined with critical review of recent studies of UHRF1 domain function, demonstrate that methyllysine recognition of LIG1 (and histone H3) by UHRF1 is not required for the maintenance of cancer cell DNA methylation patterning through cell divisions and that disruption of this function, alone, may not be a viable strategy toward antagonizing aberrant DNA methylation patterns maintained by UHRF1 in cancer cells.

## Results

### UHRF1 binds LIG1K126me2 with high affinity and selectivity over known methyllysine reader domains

**Fig. 1:**
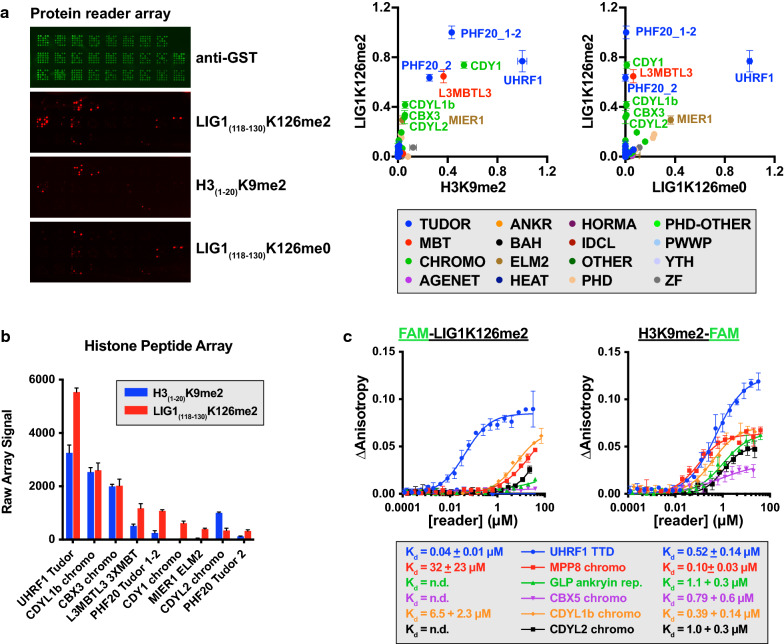
LIG1K126me2 is read by a high-affinity interaction through the UHRF1 TTD. **a** Protein reader domain microarrays consisting of 308 GST-tagged domains (see Additional file 1: Fig. S1), each printed in duplicate, were probed by either anti-GST antibody, or Cy3-labeled LIG1_(118–130)_K126me2, H3_(1–20)_K9me2, or LIG1_(118–130)_K126me0 (left). Reader arrays were quantified using ArrayNinja software. Data were normalized to the brightest signal for each peptide (right). Error bars represent the range from duplicate spots. Full reader array datasets are available in Additional file 2: Table S1. **b** The indicated GST-tagged reader domains were hybridized to histone peptide microarrays at 1 µM, followed by fluorescent detection and quantified by ArrayNinja. Results for interactions with H3_(1–20)_K9me2 and LIG1_(118–130)_K126me2 are shown. Error bars represent standard error of the mean of six printed spots. Full histone peptide array data are available in Additional file 3: Table S2. **c** Fluorescence polarization binding assays between the indicated GST-tagged reader domains and either FAM-LIG1_(118–130)_K126me2 (left) or H3_(1–20)_K9me2-FAM (right). Error bars represent the 95% confidence interval from triplicate measurements

The H3K9me2/me3-binding UHRF1 TTD was recently reported to also read LIG1K126me2 [21, 22]. We sought to determine whether this interaction was unique or whether other methyllysine reader domains compete for this non-histone interaction. Biotinylated LIG1_(118–130)_, LIG1_(118–130)_K126me0, LIG1_(118–130)_K126me2, and H3_(1–20)_K9me2 peptides were complexed with Cy5-streptavidin and hybridized to protein domain microarrays [25] that displayed 308 GST-tagged reader domains (Fig. 1a left, Additional file 1: Fig. S1). Microarrays were probed under saturating peptide concentrations in order to detect even weak interactions and provide the widest view of potential interactions. Saturation in peptide hybridization was evident by an inability to discriminate the UHRF1 TTD interaction between LIG1K126me0 and LIG1K126me2, a binding preference that was previously determined by both fluorescence polarization (FP) [21] and isothermal titration calorimetry (ITC) [22]. Further, we note that hits on the reader array that have signal >  ~ 0.7 are likely to be equivalent (i.e., saturated) and this difference is potentially due to variability in printing of the reader proteins (see anti-GST scan). Signal intensities from bound peptide were analyzed with ArrayNinja software [26] and plotted normalized to the brightest signal (Fig. 1a, right). Full datasets are reported in Additional file 2: Table S1. We observed that LIG1K126me2 interacted primarily with Tudor domains (PHD finger protein 20 (PHF20, UniProtKB Q9BVI0) and UHRF1) and chromodomains (testis-specific chromodomain protein Y 1 (CDY1, UniProtKB Q9Y6F8), chromodomain Y-like protein isoform 2 (CDYL1b, UniProtKB Q9Y232-2), chromobox protein homolog 3 (CBX3, UniProtKB Q13185), and chromodomain Y-like protein 2 (CDYL2, UniProtKB Q8N8U2)) that also bound H3K9me2 (Fig. 1a, right).

**Fig. 2:**
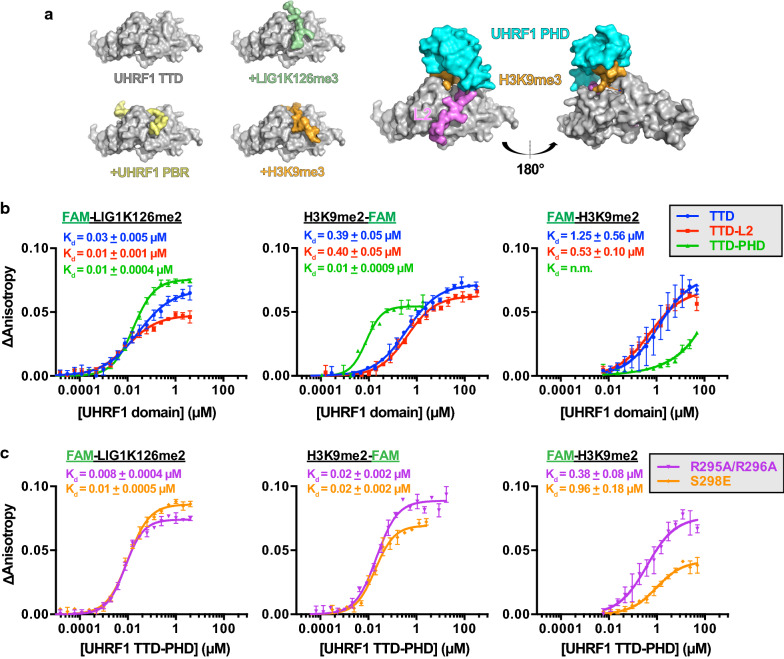
Unlike binding to H3K9me2, the UHRF1 PHD and its N-terminal linker do not modulate LIG1K126me2 recognition through the TTD. **a** Structural models of UHRF1 TTD bound to LIG1K126me3 (PDB:5YYA), UHRF1 PBR (PDB:6B9M), H3K9me3 (PDB:2L3R), and UHRF1 TTD–PHD bound to H3K9me3 (PDB:3ASK) with the linker between TTD and PHD shown in pink. **b**, **c** FP binding assays between the indicated GST-tagged **b** UHRF1 domains or **c** TTD–PHD mutants reader domains and FAM-LIG1_(118–130)_K126me2, H3_(1–20)_K9me2-FAM, or FAM-H3_(1–20)_K9me2. Error bars represent standard error of the mean from triplicate measurements

Next, we sought to validate select LIG1 hits from the protein domain microarray screen, as well as several other known H3K9 methyl readers (CBX5 chromo, MPP8 chromo, and GLP ankyrin repeats [27, 28]) with histone peptide microarrays and FP binding assays. For peptide microarrays, recombinant GST-tagged reader proteins were hybridized to streptavidin-coated glass slides printed with a library of ~ 350 biotinylated histone peptides and also LIG1K126 peptides in each methylation state (me0, me1, me2, and me3). Each candidate reader was arrayed at the same concentration (1 µM), analyzed by ArrayNinja, and raw signal intensities were plotted for six replicates (Fig. 1b). Full peptide microarray datasets are reported in Additional file 3: Table S2. We note that interactions between readers and peptides with dissociation constants > 30 µM are unlikely to be detected by peptide microarray analysis [29]. LIG1K126me2 was the top hit for the UHRF1 TTD, and unlike other readers of this mark, UHRF1 bound LIG1K126me2 with higher affinity than H3K9me2 peptides (Fig. 1b). Consistently, FP binding assays showed that the UHRF1 TTD binds LIG1K126me2 peptides with a *K*_d_ of 30–80 nM, compared to 390–520 nM for H3K9me2 (Figs. 1c, 2b). Further, other H3K9me2 readers that bind this mark in a similar affinity range as the UHRF1 TTD measured low µM or unmeasurable affinity for LIG1K126me2 (binding to LIG1K126me2 was not detected by FP for CDY1 chromodomain, PHF20 Tudors 1 and 2, or L3MBTL3 3X MBT (not shown)). In our experience, FP assays for interactions with dissociation constants >  ~ 5 µM do not reach saturation and are thus not reliably fit to non-linear regression models for *K*_d_ determination. From these data, we conclude that the UHRF1 TTD binds to LIG1K126me2 with high affinity and selectivity (the ratio of dissociation constants between H3K9me2 and LIG1K126me2 for UHRF1 is ~ 8).

### UHRF1 binds to LIG1K126me2 independently of its PHD finger

The cleft of the UHRF1 TTD interacts with positively charged peptides (Fig. 2a) including the polybasic region (PBR, amino acids ~ 644 to 655) of the same UHRF1 polypeptide [30], the linker (L2) connecting the TTD to the neighboring PHD [31], H3K9me2/me3 [32], and LIG1K126me2/me3 [22]. The UHRF1 PHD binds to the N-terminus of histone H3 and (together with L2 occupancy of the TTD cleft) promotes a multivalent *cis* conformation of the bound H3 peptide that is distinct from the conformation of H3 peptides bound to the isolated TTD (Fig. 2a, right).

We sought to determine whether the UHRF1 L2 and PHD finger participate in the UHRF1 TTD interaction with LIG1K126me2. With FP binding assays, we measured how addition of either L2 (TTD-L2), or L2 plus the PHD (TTD-PHD) affected binding of the UHRF1 TTD to LIG1K126me2 and H3K9me2. Distinct from H3K9me2, high-affinity (10–30 nM *K*_d_) interactions between UHRF1 TTD with LIG1K126me2 were maintained with inclusion of L2 or the PHD (Fig. 2b). The interaction of the UHRF1 TTD with H3K9me2 was enhanced by inclusion of the PHD, supporting the multivalent *cis* binding model of this interaction [33]. Consistently, conjugating a FAM (6-carboxyfluorescein) dye to the N-terminus of the H3 peptide blocked PHD binding (Fig. 2b, right); however, an N-terminal FAM-conjugated LIG1 peptide bound with similar affinity to the UHRF1 TTD, TTD-L2, and TTD-PHD (Fig. 2b, left). Furthermore, mutation of L2 (R295A/R296A [31]) that allowed for binding of the UHRF1 TTD-PHD to FAM-H3 and had binding constants similar to that of the TTD alone, had no effect on binding to LIG1 (Fig. 2c). The UHRF1 S298E [34] phospho-mimetic mutation in L2 is a surrogate for phosphorylation by protein kinase A (UniProtKB P17612) [35], and this mutation also had no effect on the interaction with LIG1K126me2 (Fig. 2c). These data support a mode of monovalent engagement of LIG1 by the UHRF1 TTD. Consistent with a prior structural study [22], these data also suggest that the UHRF1–LIG1K126me2 interaction is independent of the PHD and that H3K9me2 is bound by UHRF1 in a distinct manner from LIG1K126me2.

### Antagonism of UHRF1 TTD by methylated LIG1 peptides has no effect on cancer cell DNA methylation

**Fig. 3:**
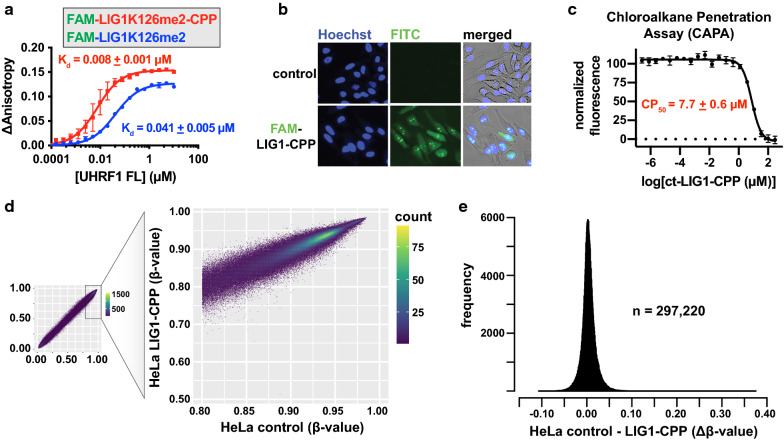
A LIG1K126me2 cell-penetrating peptide has no significant effects on HeLa cell DNA methylation. **a** Fluorescence polarization binding assays between full-length UHRF1 and FAM-labeled LIG1_(118–130)_K126me2 peptides with (red) and without (blue) a cell-penetrating peptide (CPP) sequence, -polyethylene glycol-kkkrkv. **b** Fluorescence microscopy of HeLa cells after 5-h incubation with control solvent (water) or with FAM-LIG1K126me2-CPP. **c** Chloroalkane penetration assay (CAPA) for chloroalkane-tagged (ct) LIG1K126me2-CPP in HeLa cells (CP_50_), concentration where 50% of maximal penetration was observed; error bars, the standard error from the independent experiments—three independent curve fits from three independent experiments). **d** Infinium MethylationEPIC BeadChip analysis of HeLa cells (beta values: 0, unmethylated; 1, methylated) after 7 days of incubation with control solvent or LIG1K126me2-CPP peptide at 20 µM. Scatter plots with density for all probes (left) and those that had beta values > 0.8 in control cells (right). **e** Distribution of beta-value differences (∆β) between control and LIG1K126me2-CPP-treated cells for probes in panel (**d**) that were > 0.8 in control cells (n)

As our in vitro data showed LIG1K126me2 peptides bound the UHRF1 TTD with high affinity and selectivity, we next sought to determine whether this mode of interaction might be an approach to antagonize UHRF1 function in cancer cells. We synthesized a FAM-labeled LIG1_(118–130)_K126me2 peptide with a C-terminal cell-penetrating peptide (CPP) sequence [36]. A LIG1K126me2 peptide bound recombinant full-length UHRF1 with similar affinity as the TTD and TTD-PHD, and inclusion of the CPP sequence enhanced this interaction (Figs. 2b and 3a). HeLa cells incubated with FAM-LIG1K126me2-CPP were fluorescent after 5 h of incubation with the peptide and showed diffuse and punctate staining that co-localized with the DNA-binding Hoescht dye (Fig. 3b). A similar staining pattern for FAM-LIGK126me2-CPP was observed in HCT116 cells (Additional file 4: Fig. S2A). To quantify cellular uptake, a chloroalkane penetration assay (CAPA) [37, 38] was performed. Briefly, a chloroalkane tag (ct), the covalent HaloTag ligand, was attached in place of FAM in the LIG1_(118–130)_K126me2-CPP. This ct-peptide was incubated with HeLa cells carrying a cytosolic HaloTag-GFP fusion protein. Next, cells were pulsed with ct-TAMRA to bind to the unreacted HaloTag. Fluorescence was then quantified by flow cytometry, providing an inverse relationship between fluorescence intensity and covalent ct-LIG1-HaloTag fusion. Normalized data were plotted as a function of ct-LIG1 concentration and fit to a nonlinear regression model to determine a value where 50% of the maximal penetration was achieved, or the CP_50_ (Fig. 3c). We found that the CP_50_ of ct-LIG1 was 7.7 ± 0.6 µM, similar to that of functionally active chromobox protein homolog 7 (CBX7, UniProtKB O95931) probes with ~ 100 nM in vitro *K*_d_s for their target [39], suggesting that LIG1_(118–130)_K126me2-CPP is sufficiently cell penetrant for effective UHRF1 target engagement in cells.

As both CAPA and fluorescence imaging data supported that the LIG1_(118–130)_K126me2-CPP entered cells and accessed the nuclear compartment, we sought to determine whether this high-affinity LIG1 peptide was able to antagonize UHRF1 function in cancer cells. To this end, we carried out a prolonged incubation of either vehicle control (water) or LIG1K126me2-CPP peptide with human cervix (HeLa) and colon (HCT116) adenocarcinoma cell lines (both of which have doubling times of ~ 20 h) to allow for successive rounds of replication (and detection of possible maintenance methylation defects). Cells were incubated with 20 µM LIG1K126me2 peptide, an amount above the determined CP_50_. Following 7 days of incubation with control solvent (water) or peptide, DNA methylation was measured by Illumina Methylation Epic Array, which simultaneously profiled the methylation status of ~ 850,000 CpG probes primary across promoters, enhancers, and gene bodies. There were no apparent changes observed in the distribution of beta-value densities (0, unmethylated; 1, methylated) in either HeLa or HCT116 cells (Figs. 3d, e, Additional file 4: Figure S2B), indicating that DNA methylation maintenance of bulk cell populations was not affected by these peptides.

### Depletion of LIG1 had no significant effects on DNA methylation maintenance in bulk cancer cell populations

**Fig. 4:**
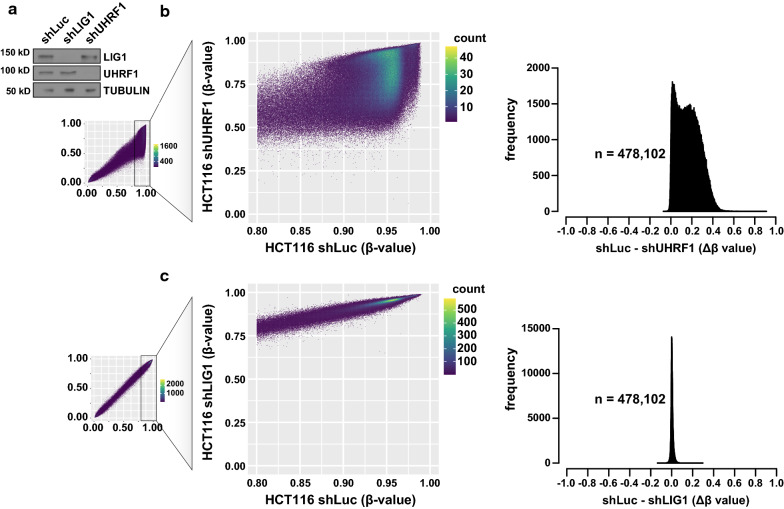
LIG1 depletion does not affect DNA methylation maintenance in HCT116. **a** Western blots confirming LIG1 or UHRF1 knockdown in HCT116 cells. **b, c** Infinium MethylationEPIC BeadChip analysis of HCT116 cells (beta values: 0, unmethylated; 1, methylated) 12 days after incorporation of shRNAs targeting UHRF1 (**b**) or LIG1 (**c**) relative to the control cell line (shLuc, shRNA targeting luciferase). Scatter plots with density for all cytosine probes (left) and those that had beta values > 0.8 in control cells (middle) are shown. Distribution of beta-value differences (∆β) between shLuc and target knockdown for probes that were > 0.8 in control cells (n) is shown (right)

LIG1 was reported to be a regulator of DNA methylation in mESCs [21]. In the aforementioned study, complete knockout of LIG1 had no effect on DNA methylation, while CRISPR-based internal deletion of 32 or 66 residues surrounding K126 of LIG1 demonstrated a reduction in DNA methylation. We depleted either LIG1 or UHRF1 from HCT116 cells by shRNA (Fig. 4a) and compared their DNA methylation profiles to control cells expressing an shRNA targeting luciferase (shLuc). After 12 days, we measured DNA methylation levels by Illumina Methylation Epic Array. While DNA methylation levels were significantly reduced following UHRF1 knockdown relative to shLuc control cells (Fig. 4b), no significant changes in the distribution of DNA methylation beta values were observed after depletion of LIG1 (Fig. 4c). These data are consistent with the absence of a DNA methylation defect after treatment with the LIG1K126me2-CPP (Fig. 3d) and further confirm that LIG1 does not contribute to UHRF1-dependent DNA methylation maintenance in bulk cancer cell populations.

### Disruption of LIG1K126- and H3K9-associated methylation has no significant effect on DNA methylation maintenance in bulk cancer cell populations

**Fig. 5:**
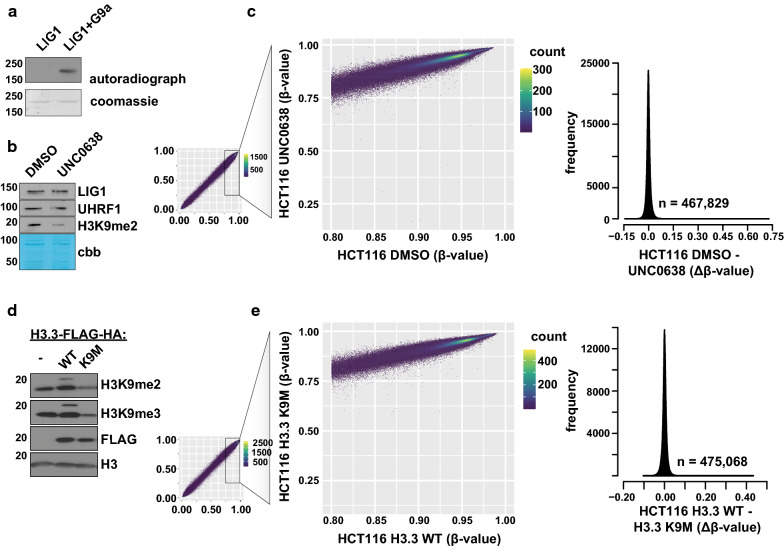
DNA methylation maintenance is stable despite global disruptions to LIG1K126- and H3K9-associated lysine methylation signaling. **a** In vitro methylation of recombinant full-length LIG1 by the catalytic domain of G9a (EHMT2). ^3^H-SAM incorporation was monitored by autoradiography and Coomassie staining is used as a loading control. **b** Western blots of HCT116 cells treated with either DMSO control or the G9a inhibitor UNC0638 (1 µM) for 48 h. cbb, Coomassie brilliant blue stain of membrane. **c **Infinium MethylationEPIC BeadChip analysis of HCT116 cells from **b**. Data are presented as in Fig. 4b, c. **d** Western blots of HCT116 cells 12 days after incorporation of lentivirus expressing wild-type H3.3 (WT) or H3.3 K9M. **e** Infinium MethylationEPIC BeadChip analysis of HCT116 cells from **d**. Data are presented as in Fig. 4b, c

We next sought to more generally determine the contribution of LIG1K126me2- and H3K9me2/me3-associated signaling to DNA methylation control. G9a and GLP are reported to be the major lysine methyltransferases for LIG1K126me2 [21] and H3K9me2 [40]. Indeed, recombinant full-length LIG1 is a substrate of G9a (Fig. 5a), and treatment of HCT116 cells with the G9a/GLP inhibitor UNC0638 effectively reduced the levels of H3K9me2 in bulk cell populations without effect on LIG1 or UHRF1 levels (Fig. 5b). EPIC array analysis of these cell populations showed no significant difference in DNA methylation patterning (Fig. 5c).

As UHRF1 is reported to bind H3K9me2 and H3K9me3, and additional lysine methyltransferases are appreciated to use H3K9 (and potentially LIG1K126) as a substrate, we sought an orthogonal approach to genetically trap lysine methyltransferases associated with these marks by stably transducing HCT116 cells with the ‘oncohistone’ mutation H3.3K9M [41, 42]. Transduction with H3.3K9M, but not wild-type H3.3, depleted H3K9me2 and H3K9me3 in these cells (Fig. 5d). Despite global depletion of H3K9me2/me3, DNA methylation profiles were largely unchanged between H3.3WT and H3.3K9M (Fig. 5e). Collectively, these data further support a model of UHRF1-dependent DNA methylation maintenance in cancer cells that does not involve histone- or non-histone methyllysine-driven interactions through the UHRF1 TTD.

### A competitive model for UHRF1, H3K9me2, and LIG1K126me2

**Fig. 6:**
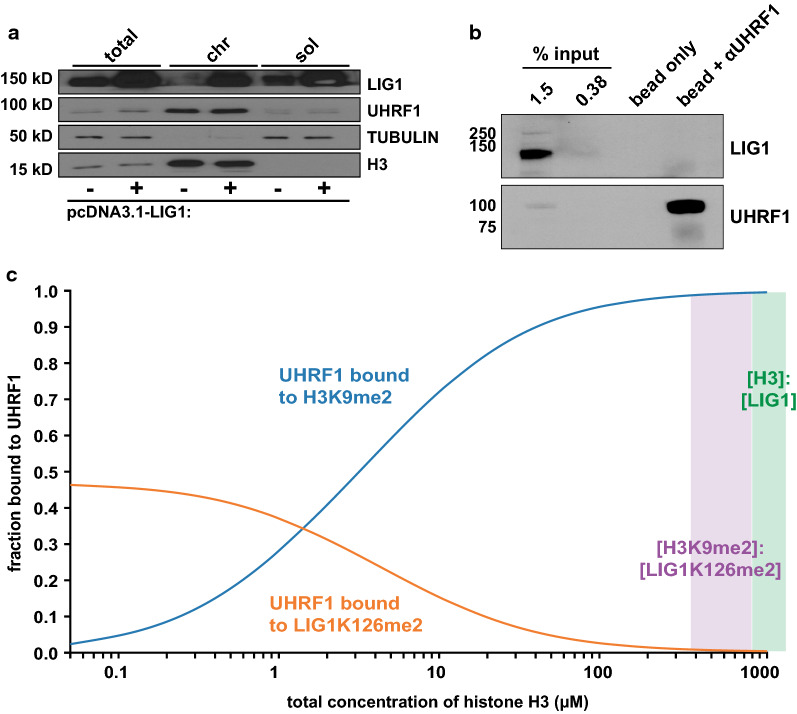
Molecular interaction analysis and theoretical modeling do not support a UHRF1–LIG1 interaction in cells. **a** Western blots for the indicated proteins following chromatin fractionation of HCT116 cells in the absence (−, empty vector control) or presence (+) of a LIG1 transgene. Total, whole cell extracts; chr, chromatin fraction; sol, soluble fraction. **b** IP of endogenous UHRF1 from HCT116 cells followed by western blot for LIG1 and UHRF1. **c** A competitive binding model for UHRF1 interactions with methylated forms of H3 and LIG1. Shaded regions show the range of approximate ratios for the methylated (purple) or unmethylated (green) binding partners of UHRF1

To begin considering other functions associated with these high-affinity UHRF1 TTD in vitro interactions, we next sought to determine the relationship between UHRF1 interactions with H3 and LIG1 in cells. We biochemically fractionated HCT116 cells between chromatin (and associated proteins) and soluble (nucleoplasm and cytoplasm) fractions with and without overexpression of full-length LIG1. In these experiments, endogenous UHRF1 was associated with chromatin fractions, while endogenous LIG1 was soluble (Fig. 6a). Furthermore, LIG1 overexpression had no observable effect on the amount of chromatin-bound UHRF1 (Fig. 6a). Given the surprising finding that endogenous UHRF1 and LIG1 were apparently not complexed after fractionation, we attempted to detect a UHRF1–LIG1 interaction via co-immunoprecipitation (co-IP) from non-fractionated HCT116 whole cell lysates (Fig. 6b). We were unable to detect LIG1 after IP with a UHRF1 antibody (Fig. 6b), and the reciprocal IP also did not show evidence of an endogenous interaction (data not shown).

In light of these findings, we hypothesized that the abundance of H3K9me2 relative to LIG1K126me2 could explain why we were unable to find evidence of a UHRF1–LIG1 interaction in cells. Many of the writers, readers, and erasers of histone PTMs are now being characterized as proteins with non-histone interactions and targets [43]. Moving forward, consideration for the vast difference in abundance of histone versus non-histone proteins in cell nuclei will be an important consideration for predicting the likelihood of non-histone protein modifications and interactions. For the case of UHRF1 and the potential for interactions with methylated forms of H3 and LIG1, we used publicly available data to generate a competitive binding model (Fig. 6c). First, quantitative proteomics data revealed that there is roughly a 1:1 ratio of UHRF1:LIG1 in a variety of cell types, and there is approximately a 1000-fold molar excess of histone H3 over LIG1 and UHRF1 [44]. Second, roughly 35% of H3 [45] and 50% of LIG1 [43] carry di-methylation at K9 and K126, respectively. Third, the *K*_*d*_s for UHRF1 for H3K9me2 and LIG1K126me2 were measured to be 0.19 µM [4] and 0.041 µM (Fig. 3a), respectively. Finally, our model assumes that only one of the proteins, H3K9me2 or LIG1K126me2, is able to interact with a UHRF1 protein through TTD-methyllysine recognition. After accounting for these parameters and using the standard mass balance and definition of binding constants, our model predicts that 99% of UHRF1 will be bound to H3K9me2 and < 1% will be bound to LIG1K126me2 (Fig. 6c). With this model and the data presented in this study, we suggest that endogenous UHRF1 and LIG1 do not directly interact at an appreciable level. Further, UHRF1 is capable of three defined contacts with chromatin (H3 N-terminus, H3K9me2/me3, and DNA), likely adding an affinity boost to chromatin, which already exists in vast excess over LIG1. These data suggest that UHRF1 and LIG1, despite maintaining high-affinity interactions in vitro, are not appreciably bound in cells.

## Discussion

In this body of work, we utilized array-based functional proteomics approaches to identify Tudor and chromodomains as the primary interactors of LIG1K126me2 (Fig. 1). The UHRF1 TTD was the tightest LIG1K126me2 interaction measured, with a dissociation constant ~ 70X tighter than the next best interaction, CDYL1b chromodomain. We further characterized the interaction between UHRF1 TTD, TTD-L2, and TTD-PHD with methylated LIG1 (Fig. 2) and concluded that the interaction is independent of the PHD domain. Despite a high-affinity in vitro interaction between UHRF1 and a LIG1K126me2 peptide, we were unable to detect changes in DNA methylation after prolonged incubation with a LIG1K126me2-CPP (Fig. 3d, e) or LIG1 knockdown (Fig. 4c). Using both chemical and genetic approaches to interfere with both histone and non-histone lysine methylation, we were unable to detect changes in DNA methylation in several cancer cell lines (Fig. 5). Further, we showed that UHRF1 and LIG1 were not complexed after chromatin fractionation, the UHRF1 chromatin interaction was not modulated by LIG1 transgene expression (Fig. 6a), and a UHRF1–LIG1 interaction was undetectable by co-IP (Fig. 6b). These data are consistent with a competitive binding model for UHRF1, H3K9me, and LIG1K126me, where the abundance of H3K9 methylation (~ 350–1000X molar excess over LIG1K126me) limits the time that UHRF1 spends bound to LIG1. We note that forced overexpression of UHRF1 and/or LIG1 will alter parameters in a competitive binding model and may drive non-physiologically relevant interactions.

Surprisingly, we were unable to perturb UHRF1-dependent DNA methylation maintenance despite treating cells with a potent, cell penetrant antagonist (LIG1K126me2 peptide) to the UHRF1 TTD, and with genetic and chemical approaches to globally deplete H3K9me2/me3 from cells. While the reading of H3K9me2/me3 by UHRF1 has been proposed as a mechanistic link between histone methylation and DNA methylation [46, 47], recent studies report minimal to no defects in DNA methylation after disruption of UHRF1 methyllysine binding function, and our studies are consistent with this interpretation. After stably expressing wild-type or Y191A/P192A TTD mutant UHRF1 in *Uhrf1* (or *Np95*) knockout mouse embryonic stem cells, the TTD mutant restored 5mC levels to 92% of the wild-type rescue as measured by high-performance liquid chromatography (HPLC) [47]. Knock-in mice homozygous for *Np95* Y187A/P188A also demonstrated a statistically insignificant reduction in global 5mC by HPLC compared to wild-type littermates, and of the 2.9 million CpGs covered by RRBS, only 0.77% were called hypomethylated in the TTD mutant relative to wild-type mice [48]. Further, in HCT116 and RKO colon cancer cell lines, simultaneous genetic knockdown/rescue assays with either wild-type or Y188A TTD mutants were indistinguishable by Illumina methylation array [20].

A recent study suggested that disruption of LIG1K126 or mutation of the UHRF1 TTD perturbed only the “replication-coupled” (less than 20 min after thymidine release) maintenance of DNA methylation, but that this defect was repaired by the end of S-phase, resulting in no measurable effect on DNA methylation in bulk cell populations [49]. The implication is that methyllysine recognition by UHRF1 may provide a slight increase in efficiency for maintenance methylation. However, given enough time (a 20- to 24-h cell cycle, for example), the DNA methylation machinery has a mechanism to repair this disruption before the cell divides. This intriguing new finding may help to explain our findings as well as those of the aforementioned studies. Our studies and these above-referenced findings also call into question a recent report implicating H3K9me3 recognition directly by DNMT1 as a mechanism that contributes to its DNA methylation maintenance function [50].

These previous studies, although focused on methyllysine recognition by UHRF1 in the context of histones, clearly demonstrate that a functional UHRF1 TTD, and therefore methyllysine recognition, is dispensable for DNA methylation maintenance. We therefore conclude that neither H3K9me nor LIG1K126me2 interaction with UHRF1 are required for the maintenance of bulk DNA methylation. However, we consider the possibility that our approaches may miss DNA methylation changes that occur transiently (e.g., during the first 20 min of S-phase). Rather, N-terminal H3 recognition through the UHRF1 PHD, independent of H3K9 methylation, is required for histone ubiquitination [12] and DNA methylation maintenance [20]. It is interesting to note that a free N-terminus of histone H3 also contributes to de novo DNA methylation through the PHD-like ADD domain [51] of DNA (cytosine-5)-methyltransferase 3A (DNMT3A, UniProtKB Q9Y6K1) [52, 53], further emphasizing the importance of the H3 tail in DNA methylation control.

So, what is the functional significance of the interaction between UHRF1 and LIG1? Our competitive binding model leads us to speculate that very high-affinity interactions, as in the case of UHRF1 TTD-LIG1K126me2, are not necessarily meaningful in an abundant sea of high-affinity histone interactions with UHRF1 (Fig. 6). Collectively, our data and the studies noted above suggest that the UHRF1 TTD is not a viable pharmacologic target to directly block chromatin interactions and reduce DNA methylation. However, we suggest that the high affinity and apparent specificity among H3K9me readers between LIG1K126me2 and the UHRF1 TTD may serve as a basis for chemical probes to study cellular functions associated with the UHRF1 TTD and for the target-specific end of bivalent chemical degraders [54, 55].

## Materials and methods

### Protein and peptide array experiments

A collection of 308 GST recombinant fusion proteins were expressed and purified using Glutathione Sepharose beads (GE Healthcare) as described [56, 57]. The GST-tagged methyllysine reader domains were arrayed onto nitrocellulose-coated glass slides (Oncyte Avid slides, Grace Bio-Labs) using a pin arrayer (Aushon). GST proteins were printed in duplicate as indicated in Additional file 1: Fig. S1. C-terminal biotinylated LIG1_(118–130)_K126me0, LIG1_(118–130)_K126me0, or H3_(1–20)_K9me2 peptides were fluorescently labeled with Cy3-streptavidin and then 100 µg of each peptide was hybridized on the microarrays in 1.8 mL of PBST with 3% milk and 3% bovine serum albumin. Binding of fluorescent peptides was visualized using a GenePix 4200A Microarray Scanner (Molecular Devices), scanned at 10 µm resolution, and quantified using ArrayNinja [26]. Data shown are normalized within each peptide dataset to the brightest signal. Histone peptide microarray experiments were performed as described [4], with the following exceptions: proteins were hybridized at 1 µM for 1 h and all hybridization steps were performed at room temperature. Raw data for both reader and histone peptide microarrays are available in Additional file 2: Table S1 and Additional file 3: Table S2.

### Fluorescence polarization binding assays

Fluorescence polarization binding assays were performed as described [4] and plotted as change in anisotropy. Protein residues correspond to UniProt numbering: UHRF1 TTD (a.a. 125–285), UHRF1 TTD-L2 (a.a. 125–301), UHRF1 TTD–PHD (a.a. 125–364), UHRF1 full-length (a.a. 1–793). Isolated domains were characterized as GST fusions and full-length protein as an MBP fusion. FAM-H3_(1–20)_K9me2, H3_(1–20)_K9me2-FAM, and FAM-LIG1_(118–130)_K126me2 were purchased from GenScript.

### Peptide synthesis

The fluorescent peptide FAM-LIG1_(118–130)_K126me2-PEG-CPP (5-FAM-IPKRRTARK(Me_2_)QLPK-PEG-kkkrkv-NH_2_, where PEG stands for a short polyethylene glycol linker and kkkrkv for a cell-penetrating peptide sequence [36], containing all D-amino acids to improve proteolytic stability, was synthesized on a PTI Symphony peptide synthesizer using Fmoc solid-phase synthesis with HATU as a coupling reagent. 5-FAM group was introduced at the end of synthesis by 5 h coupling with 5-FAM-OH and DIPC/HOAt/DIEA in DMF. The peptide for CAPA (chloroalkane tag-IPKRRTARK(Me_3_)QLPK-kkkrkv-NH_2_) was synthesized on a CEM Liberty Blue peptide synthesizer using microwave-assisted Fmoc-chemistry with CarboMAX coupling [58]. A chloroalkane tag (ct) was introduced at the end of synthesis by a 1 h coupling with chloroalkane tag (carboxylic acid form) and PyAOP/HOAt/DIEA in DMF. All peptides were synthesized using ChemMatrix Rink amide resin (loading 0.48 meq/g), cleaved from the resin and deprotected by 2-h incubation with 2.5% water, 2.5% TIS in TFA, precipitated from cold diethyl ether, washed 3 times with ether, air dried, dissolved in 50% acetonitrile, and lyophilized. The crude peptides were purified by preparative RP-HPLC and lyophilized. The purified peptides were analyzed by analytical RP-HPLC and MALDI-MS.

### Cell culture and DNA methylation analysis

HCT116 colon cancer cells were maintained in McCoy’s 5A media (Gibco) and HeLa cervical cancer cells in DMEM (Gibco), each with 10% fetal bovine serum (Sigma) without antibiotics at 37 °C in 5% CO_2_. Cells were maintained at densities of 20–90% and split every 2–3 days to avoid contact inhibition. LIG1_(118–130)_K126me2-CPP peptides were incubated with cells at a final concentration of 20 µM in 1.5 mL in 6-well plates for 7 days. HCT116 cells were treated with either DMSO or UNC0638 (1 µM) for 48 h in 10-cm dishes prior to DNA methylation analysis. DNA was extracted by DNEasy kit (Qiagen), treated with RNAse (A/T1), and a DNA precipitation was performed. For knockdown studies with shRNA against UHRF1 (pLKO-PGK-PuroR, TRCN0000273256), LIG1 (pLKO-PGK-PuroR, TRCN0000048495), or luciferase (pLKO-PGK-PuroR, TRCN0000072246) and overexpression of H3.3 WT or K9M (pCDH-EF1-H3.3-FLAG-HA-ires-Puro, a gift from Dr. Peter Lewis), lentivirus was generated in HEK293T by standard protocol (Addgene). HCT116 cells were transduced in the presence of polybrene (8 µg/mL), selected by puromycin (2 µg/mL for 2 days for pLKO.1, or 4 µg/mL for the duration with pCDH), and maintained in culture for a total of 12 days before harvesting for western blot and DNA methylation analysis. Extracted DNA was provided to the Van Andel Institute Genomics Core for Infinium Methylation EPIC BeadChip analysis. Raw.idat files (available at NCBI GEO: GSE147518, GSE148086) were converted to methylation beta values in R by the command openSesame [59]. Density scatter plots were visualized with the command geom_hex with 500 bins in the R package ggplot2 [60], delta beta distributions were visualized with the command hist with 1000 breaks, and mean and median were calculated with the summary command in R.

### Chloroalkane penetration assay (CAPA) and cell-penetrating peptide visualization

CAPA data were acquired as previously described [38], with compound dilutions and control samples prepared on the day of the experiment. For imaging of FAM-LIG1_(118–130)_K126me2-CPP, HeLa or HCT116 cells were plated in Lab-Tek Chamber Slides. The next day, cells were incubated with either 10 μM peptide or water in 500 μL of their respective media for 5 h. Cells were washed 1× with PBS, fixed in 4% formaldehyde in PBS for 5 min at room temperature, washed 2× with PBS, incubated with 6 μM Hoechst (Thermo Fisher 33342) in PBS for 5 min at room temperature, followed by 1 wash with PBS. Cells were mounted in 20 mM Tris pH 8.0 with 50% glycerol and imaged on an EVOS FLoid microscope. Control- and peptide-treated cells were imaged with the same settings and all images were handled identically between control and treated cells.

### In vitro lysine methyltransferase assays

Recombinant G9a (EHMT2) catalytic domain was prepared as described in a previous study [61]. Recombinant human LIG1 was generated by cloning the full-length cDNA (residues 1–919, acquired from DNASU) into a modified pQE vector with an N-terminal 6×-His-maltose binding protein (MBP) tag. LIG1 was expressed and purified as previously described for UHRF1 in the same vector [4]. One µg of tagged LIG1 and 1 µg of G9a were combined in 10 µL of reaction buffer (50 mM Tris, pH 8.8, 5 mM MgCl_2_, 4 mM DTT) and incubated with 1 µCi (1 µL at 66 µM) of ^3^H-SAM for 30 min at room temperature. The reaction was quenched and visualized as described [61].

### Chromatin association assay

Harvested cells were resuspended in 120 µL cold CSK buffer [10 mM PIPES pH 7.0, 300 mM sucrose, 100 mM NaCl, 3 mM MgCl_2_, 0.1% Triton X-100, protease inhibitors (Roche; 1 tablet per 20 mL)]. Cells were kept on ice for 20 min. Total protein was quantified by Bradford assay (BioRad), and 10% was combined with an equivalent volume of cold endonuclease-supplemented CSK (Pierce 88702, 250 units/5 mL). Remaining cell lysates were centrifuged at 1300*g* for 5 min at 4 °C. The supernatant (soluble fraction) was collected. The chromatin pellet was washed 1× in CSK buffer, and pelleted at 1300*g* for 5 min at 4 °C. The chromatin pellet was solubilized in cold endonuclease-supplemented CSK buffer. Chromatin association assays were performed 24 h after the following transfections. For overexpression of LIG1 (UniProtKB P18858), cDNA was obtained from DNASU and cloned by Gibson assembly into pcDNA3.1 without a tag. HCT116 cells (6-well dish) were transfected with either 2 µg pcDNA3.1-LIG1 or empty pcDNA3.1 (−) with 1:3 ratio of XtremeGene HP (Roche) transfection reagent.

### Co-immunoprecipitation

HCT116 cells were grown to ~ 80–90% confluence in a 10-cm dish and harvested by scraping into cold PBS. After collection by centrifugation at 300 rcf for 5 min, cells were suspended in 450 µL of CSK buffer with 1 tablet of protease inhibitor (Roche, Complete Mini), 1 tablet of phosphatase inhibitors (Roche, PhosSTOP), and 1 µL of nuclease (Pierce 88702) per 5 mL of CSK. Cells were kept on ice for 20 min, followed by five passages through a 27 gauge needle. Lysates were cleared by centrifugation at max speed (benchtop centrifuge) at 4 °C for 5 min. Soluble material was quantified by Bradford Assay (BioRad) and 300 µg was added to either 4 µL of anti-UHRF1 antibody (CST 12387, 1 mg/mL) or 4 µL of CSK buffer (bead only) and brought to a final volume of 200 µL. Lysates and antibody were incubated overnight at 4 °C with rotation. The next morning, lysates were added to 25 µL of Protein A-coated beads (Invitrogen, 1001D) that were washed 1× in CSK buffer. Complexes were incubated with beads for 30 min at room temperature with rotation. Beads were washed 2× with 500 µL of CSK buffer and then boiled in 30 µL of 1× SDS loading buffer. Twenty-five µL of eluted material was loaded onto gels for SDS-PAGE and western blots. Indicated inputs were from 300 µg (1.5% or 0.38%).

### Western blotting

For western blots, cells were lysed in cold CSK buffer. Lysates were quantified by Bradford Assay (BioRad) and for Fig. 4a, 10 µg total protein was loaded for LIG1 and UHRF1, while 1 µg was loaded for Tubulin. For Fig. 5b, 10 µg of protein was loaded for LIG1 and UHRF1, while 2 µg was loaded for H3K9me2. For Fig. 5d, 2.75 µg of protein was loaded for all blots. For Fig. 6a, 8.2 µg of protein was loaded for LIG1 and UHRF1, while 0.75 µg was loaded for Tubulin and H3. Antibodies and dilutions used were as follows: LIG1 (ProteinTech 18051–1-AP, 1:1000, lot # unk.), UHRF1 (Cell Signaling 12387, 1:1000, lot # 1), FLAG (Sigma 1804, 1:5000, lot # unk), beta 3 Tubulin (UniProtKB Q13509, ProteinTech 66240-1-Ig, 1:100,000, lot # unk.), histone H3 (EpiCypher 13–0001, 1:100,000, lot # 12320001), H3K9me2 (Abcam 1220, 1:5000, lot # unk.), and H3K9me3 (Active Motif 39161, 1:5000, lot # unk). In Figs. 5d and 6a, 1:50,000 dilutions were used for Tubulin or H3 antibodies. All blotting procedures were carried out in PBS with 5% BSA and 0.1% Tween. Secondary antibodies (GE Life Sciences) were anti-rabbit (1:10,000) or anti-mouse (1:5000) conjugated to horseradish peroxidase.

### Competitive binding model for UHRF1, H3K9me2, and LIG1K126me2

For the competitive binding model, we used the standard mass balance and definition of binding constants. The plot in Fig. 6 is the solution to the following equation:

$${UHRF1}^{t}={UHRF1}^{f}+\left[UHRF1 H3K9me2\right]+[UHRF1 LIG1K126me2]$$

subject to

$${H3K9me2}^{t}={H3K9me2}^{f}+\left[UHRF1 H3K9me2\right]$$

$${LIG1K126me2}^{t}={LIG1K126me2}^{f}+\left[UHRF1 LIG1K126me2\right],$$

where

$${K}_{B}=\frac{[X Y]}{{X}^{f}{Y}^{f}}, t=total, f=free.$$

## Supplementary information

**Additional file 1: Figure S1.** Methyl reader domain array layout corresponding to images in Fig. 1a.

**Additional file 2: Table S1.** Complete methyl reader domain microarray data as quantified by ArrayNinja software.

**Additional file 3: Table S2.** Complete histone peptide microarray data corresponding to Fig. 1b.

**Additional file 4: Figure S2.** A LIG1K126me2 cell penetrating peptide has no significant effects on HCT116 cell DNA methylation. **(A) **Fluorescence microscopy of HCT116 cells after 5-h incubation with control solvent (water) or with FAM-LIG1K126me2-CPP. **(B)** Infinium MethylationEPIC BeadChip analysis of HCT116 cells (beta values: 0, unmethylated; 1, methylated) after 7 days of incubation with water (control) or LIG1K126me2-CPP peptide at 20 µM. Scatter plots with density for all probes (left), those that had beta value > 0.8 in control cells (middle), and distribution of ∆β (right) between control and LIG1K126me2-CPP treated cells for probes that were > 0.8 in control cells (n).

## Data Availability

Methylation Epic Array: Gene Expression Omnibus GSE147518 (https://www.ncbi.nlm.nih.gov/geo/query/acc.cgi?acc=GSE147518). Methylation Epic Array: Gene Expression Omnibus GSE148086 (https://www.ncbi.nlm.nih.gov/geo/query/acc.cgi?acc=GSE148086). ArrayNinja software: https://research.vai.org/Tools/arrayninja/. All plasmids/constructs available on request.
