# Supplementary material for: The histone and non-histone methyllysine reader activities of the UHRF1 tandem Tudor domain are dispensable for the propagation of aberrant DNA methylation patterning in cancer cells
Source: Epigenetics Chromatin. 2020 Oct 23;13:44. doi: 10.1186/s13072-020-00366-4 (PMC7585203; doi:10.1186/s13072-020-00366-4)
Supplement: Supplementary file 1 — Additional file 1: Figure S1. Methyl reader domain array layout corresponding to images in Fig. 1a. [file 13072_2020_366_MOESM1_ESM.pdf]

Vaughan et al, Fig. S1

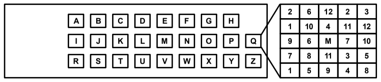

Methyl Reader Domain Array

Block A
TUDOR
1) TDRD1-2 [NP_942090.1]
2) TDRD1-3 [NP_942090.1]
3) TDRD1-4 [NP_942090.1]
4) TDRD2/TDRKH [NP_006853.2]
5) TDRD3 [NP_110421.1]
6) TDRD4-1/RNF17 [NP_112567.2]
7) TDRD4-3/RNF17 [NP_112567.2]
8) TDRD4-4/RNF17 [NP_112567.2]
9) TDRD5 [NP_001186014.1]
10) TDRD6-5 [NP_001161831.1]
11) TDRD6-6 [NP_001161831.1]
12) TDRD6-7 [NP_001161831.1]

Block B
TUDOR
1) TDRD8 [NP_001247434.1]
2) TDRD9 [NP_694591.2]
3) TDRD10 [NP_001091945.1]
4) TDRD11/SND1/p100TSN [NP_055205.2]
5) TDRD12-1 [NP_001104292.1]
6) 53BP1(1-2) [NP_005648.1]
7) AKAP1 [NP_003479.1]
8) ARID4A [NP_002883.3]
9) ARID4B [NP_001193723.1]
10) JMJD2A(1-2) [NP_055478.2]
11) LIN9 [NP_775106.2]
12) Pombe1 [CAA22823]

Block C
TUDOR
1) PHF1 [NM_002636.4]
2) PHF19 [NM_001286840.1]
3) PHF20 [NP_057520.2]
4) PHF20-2 [NP_057520.2]
5) PHF20L1 [NP_057102.4]
6) SETDB1 [NP_001138887.1]
7) SGF29 [NP_612423.1]
8) SMN [NP_000335.1]
9) SPF30/SMNDC1 [NP_005862.1]
10) UHRF1 [NP_001041666.1]
11) ZGPAT [NP_115916.3]

Block D
TUDOR+PHD
1) JMJD2B/KDM4B [AA144293.1]
2) JMJD2C/KDM4C [NP_055876.2]
3) MTF2 [NP_001157864.1]
MBT
4) L3MBTL1(1-3) [NP_056293.4]
5) L3MBTL3 [NP_115814.1]
BAH
6) ORC1 [NP_001177747.1]
TUDOR
7) SPIN1 FL [Q9Y657]
8) SPIN2A FL [Q99865]
9) SPIN2B FL [Q9BPZ2]
10) SPIN3 FL [Q5JUX0]
11) SPIN4 FL [Q56A73]

Block E
AGENET
1) FMR1 [NP_002015.1]
2) FXR1 [NP_005078.2]
3) FXR2 [NP_004851.2]
AGENET+KH
4) FMRP [Q06787.1]
ANK
5) GLP [NP_079033.4]
6) IkB FL
HEAT
7) N-CAPD3(1-4) [NP_056076.1]
8) N-CAPD3(5-8) [NP_056076.1]
OTHER
9) CYB5B FL [NP_085056.2]
10) FKBP12 FL [AAA58476]
11) MeCP2

Block F
HORMA
1) HORMAD1 [Q86X24]
2) HORMAD2 [Q8N7B1]
3) MAD2L1 [Q13257]
4) MAD2L2 [Q9UI95]
5) hMAD2 WT (134) [NM_002358.3] *
YTH
6) YTHDC1 [Q96MU7]
7) YTHDC2 [Q9H6S0]
8) YTHDF1 [Q9BYJ9]
9) YTHDF2 [Q9Y5A9]
10) YTHDF3 [Q7Z739]
IDCL
11) PCNA [NP_872590.1]

Block G
PWWP
1) BRD1
2) BRPF3
3) DNMT3A
4) DNMT3B
5) HDGF
6) HDGF2/HDGFRP2
7) HDGFL1
8) HDGFRP3
9) LEDGF/PSIP1
10) MSH6
11) MUM1L1
12) MBD5

Block H
PWWP
1) N-PAC/GLYR1
2) NSD1
3) Peregrin/BRPF1
4) PWWP2/PWWP2B
5) PWWP2A
6) WHSC1/MMSET/NSD2
7) WHSC1L1/NSD3

Block I
CHROMO
1) HP1b/CBX1 [NP_006798.1]
2) CBX2 [NP_005180.1]
3) HP1g/CBX3 [NP_009207.2]
4) CBX4 [NP_003646.2]
5) HP1a/CBX5 [NP_001120793.1]
6) CBX6 [NP_055107.3]
7) CBX7 [NP_783640.1]
8) CBX8 [NP_065700.1]
9) CDY1 [NP_733841.1]
10) CDYL1b [AAI08726.1]
11) CDYL2 [BAC04720.1]
12) MSL3 [NP_523353.2]

Block J
CHROMO
1) ARID4A [NP_002883.3]
2) ARID4B [NP_057458.4]
3) MPP8 [NP_059990.2]
4) MRG15 [NP_006782.1]
5) MYST1/MOF [NP_892003.2]
6) SMARCC1 [NP_003065.3]
7) SMARCC2 [NP_003066.2]
8) SUV39H1 [NP_003164.1]
9) SUV39H2 [NP_1180353.1]
10) TIP60 [NP_874369.1]

Block K
CHROMO
1) CHD1 [NP_001261.2]
2) CHD2 [NP_001262.3]
3) CHD6 [NP_115597.3]
4) CHD7 [NP_060250.2]
5) CHD8 [NP_1164100.1]
6) CHD9 [NP_079410.4]
CHROMO+PHD
7) CHD3 [Q12873]
8) CHD4 [Q14839]
9) CHD5 [Q8TDI0]

Block L
PHD
1) ASXL1
2) ASXL2
3) ASXL3
4) TAF3
5) DATF1/DIDO1
6) RAG2
7) RSF1/HBXAP
8) SHPRH
9) TCF19
PHD(2)
10) AIRE
11) MTF2

Block M
PHD
1) ING1
2) ING2
3) ING3
4) ING4
5) ING5
6) PYGO1
7) PYGO2
8) UHRF1
9) UHRF2
PHD(2)
10) KAT6A/MYST3
11) KAT6B/MYST4

Block N
PHD
1) KDM5A(1)
2) KDM5A(2)
3) KDM5A(3)
4) KDM5B(1)
5) KDM5B(2)
6) KDM5B(3)
7) KDM5C(1)
8) KDM5C(2)
9) KDM5D(1)
10) KDM5D(2)
11) KDM7A

Block O
PHD
1) NSD1(4)
2) WHSC1/MMSET/NSD2(4)
3) WHSC1L1/NSD3(4)
PHD(2)
4) NSD1(1-2)
5) WHSC1/MMSET/NSD2(1-2)
6) WHSC1L1/NSD3(1-2)
PHD+PWWP
7) NSD1(3)
8) WHSC1/MMSET/NSD2(3)
9) WHSC1L1/NSD3(3)

Block P
PHD
1) ASH1L
PHD-BAH
2) ASH1L
BAH
3) BAHC1
4) BAHD1
5) DNMT1(1)
6) DNMT1(2)
7) ORC1

Block Q
ELM2
1) EMSA1
2) MIER1
3) MIER2
4) MIER3
5) RCOR1
6) RCOR2
7) RCOR3
8) MTA1
9) MTA2
10) MTA3
11) RERE

Block R
PHD+BROMO+BAH
1) ASH1L(long)
PHD+BROMO+PWWP
2) ZMYND8/RACK7
3) ZMYND11
PHD+BROMO+SAND
4) SP100
5) SP110
6) SP140 [Q13342]
7) SP140L [Q9H930]
PHD+DDT+WHIM1
8) BPTF/FALZ(1)
PHD+BROMO
9) FALZ/BPTF(2)

Block S
PHD
1) BAZ1A [Q9NRL2]
2) TRIM28 [Q13263]
PHD+BROMO
3) BAZ1B [Q9UIG0]
4) BAZ2A [Q9UIF9]
5) BAZ2B [Q9UIF8]
6) TRIM24 [O15164]
7) TRIM28 [Q13263]
8) TRIM33/TIF1G [Q9UPN9]
9) TRIM66 [O15016]
10) CBP/CREBBP [Q92793]
11) p300/EP300 [Q09472]

Block T
PHD
1) PHF2 [O75151]
2) PHF3 [Q92576]
3) PHF8/JHDM1F [Q9UPP1]
4) PHF12(1) [Q96QT6]
5) PHF12(2) [Q96QT6]
6) PHF13 [Q86YI8]
7) CXXC1/PHF18 [Q9P0U4]
8) PHF20 [Q9BVI0]
9) PHF20L1 [A8MW92]
10) PHF22/INTS12 [Q96CB8]
11) PHF23 [Q9BUL5]
12) PHF24 [Q9UPV7]

Block U
PHD
1) PHF14(3) [O94880]
2) PHF21B [Q96EK2]
PHD(2)
3) PHF1 [O43189]
4) PHF10 [Q8WUB8]
5) PHF19 [Q5T6S3]
PHD+ZF-C2HC
6) PHF14(1-2) [O94880]
7) PHF15/JADE2 [Q9NQC1]
8) PHF16/JADE3 [Q92613]
9) PHF17/JADE1 [Q6IE81]
PHD+AT hook
10) PHF21A [Q96BD5]

Block V
PHD+C2HC
1) BRPF1/Peregrin [P55201]
2) BRPF3 [Q9ULD4]
3) KMT2A/MLL1(4) [Q03164]
4) KMT2B/MLL2(4) [Q9UMN6]
5) KMT2C/MLL3(1-4) [Q8NEZ4]
6) KMT2C/MLL3(8) [Q8NEZ4]
7) KMT2D/MLL4(1-3) [O14686]
8) KMT2D/MLL4(7) [O14686]
9) MLLT6 [P55198]
10) MLLT10 [P55197]

Block W
PHD (ring)
1) DTX4 [Q9Y2E6]
PHD(2)
2) DPF1 [Q92782]
3) DPF3 [Q92784]
PHD(2)+C2H2
4) DPF2 [Q92785]
5) DPF3 [Q92784]
PHD+C2H2+PHD(2)
6) BRD1 [O95696]
PHD
7) KMT2A/MLL1(1-3) [Q03164]
8) KMT2B/MLL2(1-3) [Q9UMN6]
9) KMT2C/MLL3(5-7) [Q8NEZ4]
10) KMT2D/MLL4(4-6) [O14686]
11) KMT2E/MLL5 [Q8IZD2]

Block X
ZF-CW
1) KDM1B/AOF1/LSD2 [Q8NB78]
2) MORC1 [Q86VD1]
3) MORC2 [Q9Y6X9]
4) MORC3 [Q14149]
5) MORC4 [Q8TE76]
ZF-CW+PWWP
6) ZCWPW1 [Q9H0M4]
7) ZCWPW2 [Q504Y3]
ZF-ring+PHD
8) PHRF1 [Q9P1Y6]
ZF-CXXC+PHD
9) FBXL19 [Q6PCT2]

Block Y
BAH
1) MTA1
2) MTA2
3) MTA3
4) RERE
5) TNC18
BAH-ELM2
6) MTA1
7) MTA2
8) MTA3
9) RERE
ELM2
10) TREF1
11) ZN541

Block Z
ELM2-SANT
1) EMSA1
2) MIER1
3) MIER2
4) MIER3
5) RCOR1
6) RCOR2
7) RCOR3
8) TREF1
9) ZN541
10) RERE
BAH-ELM2-SANT
11) RERE
