# Supplementary figures and images for: The histone and non-histone methyllysine reader activities of the UHRF1 tandem Tudor domain are dispensable for the propagation of aberrant DNA methylation patterning in cancer cells
Source: Epigenetics Chromatin. 2020 Oct 23;13:44. doi: 10.1186/s13072-020-00366-4 (PMC7585203; doi:10.1186/s13072-020-00366-4)

A)

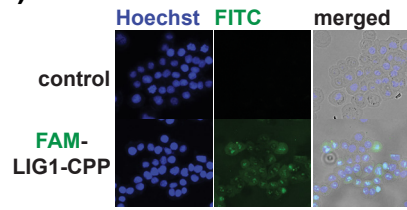

B)

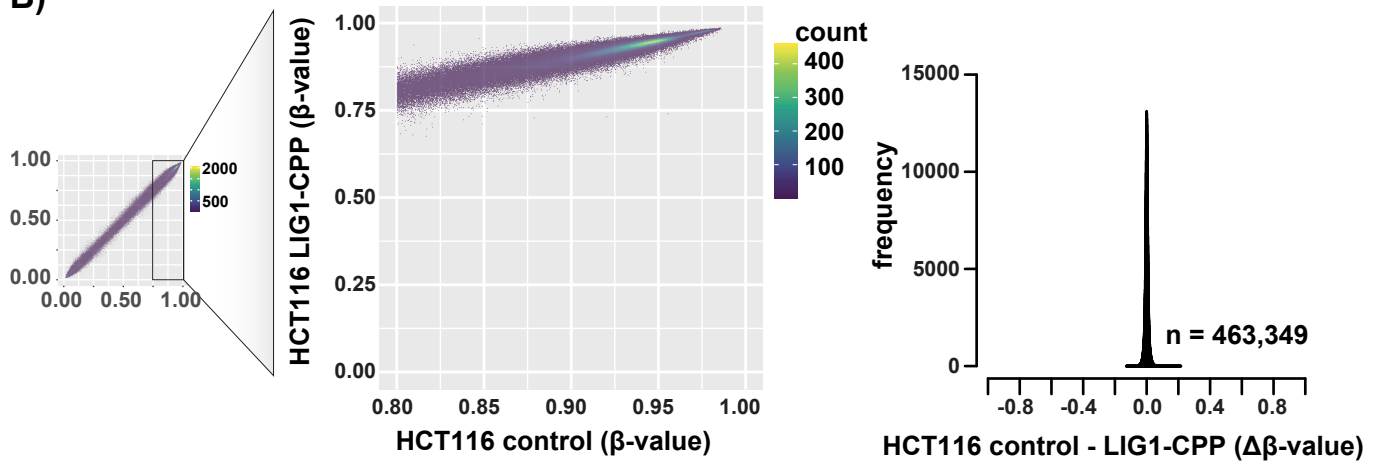

Supplement: Supplementary file 4 — Additional file 4: Figure S2. A LIG1K126me2 cell penetrating peptide has no significant effects on HCT116 cell DNA methylation. (A) Fluorescence microscopy of HCT116 cells after 5-h incubation with control solvent (water) or with FAM-LIG1K126me2-CPP. (B) Infinium MethylationEPIC BeadChip analysis of HCT116 cells (beta values: 0, unmethylated; 1, methylated) after 7 days of incubation with water (control) or LIG1K126me2-CPP peptide at 20 µM. Scatter plots with density for all probes (left), those that had beta value > 0.8 in control cells (middle), and distribution of ∆β (right) between control and LIG1K126me2-CPP treated cells for probes that were > 0.8 in control cells (n). [file 13072_2020_366_MOESM4_ESM.pdf]
